# Scalable 18,650 aqueous-based supercapacitors using hydrophobicity concept of anti-corrosion graphite passivation layer

**DOI:** 10.1038/s41598-021-92597-y

**Published:** 2021-06-22

**Authors:** Praeploy Chomkhuntod, Pawin Iamprasertkun, Poramane Chiochan, Phansiri Suktha, Montree Sawangphruk

**Affiliations:** 1 Centre of Excellence for Energy Storage Technology (CEST), Department of Chemical and Biomolecular Engineering, School of Energy Science and Engineering, Vidyasirimedhi Institute of Science and Technology, Rayong, 21210 Thailand; 2 Department of Applied Physics, Faculty of Sciences and Liberal Arts, Rajamangala University of Technology Isan, Nakhon Ratchasima, 30000 Thailand

**Keywords:** Supercapacitors, Corrosion

## Abstract

Scalable aqueous-based supercapacitors are ideal as future energy storage technologies due to their great safety, low cost, and environmental friendliness. However, the corrosion of metal current collectors e.g., aluminium (Al) foil in aqueous solutions limits their practical applications. In this work, we demonstrate a low-cost, scalable, and simple method to prepare an anti-corrosion current collector using a concept of hydrophobicity by coating the hydrophobic graphite passivation layer on the Al foil via a roll-to-roll coating technology at the semi-automation scale of production pilot plant of 18,650 cylindrical supercapacitor cells. All qualities of materials, electrodes, and production process are therefore in the quality control as the same level of commercial supercapacitors. In addition, the effects of the graphite coating layer have been fundamentally evaluated. We have found that the graphite-coated layer can improve the interfacial contact without air void space between the activated carbon active material layer and the Al foil current collector. Importantly, it can suppress the corrosion and the formation of resistive oxide film resulting in better rate capability and excellent cycling stability without capacitance loss after long cycling. The scalable supercapacitor prototypes here in this work may pave the way to practical 18,650 supercapacitors for sustainable energy storage systems in the future.

## Introduction

Nowadays, energy storage systems have been attracted attention due to the increasing demand for electrical power supplies used in portable devices, electric vehicles, as well as smart power grids. Supercapacitors are potential candidates for energy storage systems due to their high power delivery capability and great cycling stability^1^. Among various electrolyte systems, aqueous-based electrolytes have been attractive due to their non-flammability along with non-toxicity^2^. In addition, they provide a higher power density (> 10 kW kg^−1^) than those using organic electrolytes^3^, ionic liquids^4^, and water-in-salt electrolytes due to their fast ionic transport properties^5,6^. However, one of the key challenges limiting practical use of aqueous-based supercapacitors is a corrosion issue of metal current collectors in aqueous-based electrolytes. Hence, corrosion-resistant materials such as platinum, stainless steel, and nickel are needed to prevent the corrosion of current collectors in aqueous solutions. However, these materials have high cost, resulting in the limitation of large-scale production^7^. For this aspect, the modifications of low-cost current collectors have been made tremendous efforts. The aluminium (Al) foil is an alternative current collector due to its low cost and lightweight^7,8^. Unfortunately, the Al foil can be corroded in aqueous solutions by a formation of aluminium oxide (Al_2_O_3_) film as a resistive passivation layer that leads to the low conductivity of a current collector^9^. This resistive oxide layer is instantaneously formed as a uniform and amorphous layer with a thickness of ∼2–10 nm on Al foil surface^10^.

Recently, the surface modifications of metal current collectors with carbon-based materials (i.e., graphite^11,12^, graphene^13,14^, and carbon nanotubes^15^) have attracted great attention to improve the physical properties and electrochemical performance of the modified electrodes for various energy storage systems. Those publications show remarkable improvements of the modified electrodes including suppression of corrosion^13^, improvement of interfacial contact^14^, and reduction of internal resistance^16^. For this prospect, a graphite-coated aluminium current collector might be a potential candidate as a low-cost and anti-corrosion current collector for aqueous supercapacitors since graphite is an abundant material and low production cost, as well as, it provides great chemical stability and high electrical conductivity^17^. Although there is commercial carbon-coated aluminium foil available, it is not compatible with aqueous-based electrolytes because it composes of water-based binder which can be dissolved in aqueous solutions, leading to the peeling of carbon-coated layer. Moreover, there are few studies on the fundamental aspects of the properties and electrochemistry of the modified current collectors. Thus, the fundamental investigation of the graphite passivation layer fabricated via a roll-to-roll coating technology at the semi-automation level of the production pilot with high quality and reliability on the current collector to the performance of energy storage is needed.

In this work, we demonstrate a simple and low-cost method to modify a corrosion-resistant current collector. The carbon coating recipe of both activated carbon and graphite on aluminium foils has followed the previous report by JH Lee et al. They prepared the activated carbon coated Al foil as a cathode for a hybrid supercapacitor^18^. The graphite passivation layer was prepared by the roll-to-roll coating method with a finely tuned thickness of 25 μm on Al current collector. The hydrophobicity from a high surface roughness of graphite-coated results in an anti-corrosion behaviour of the as-modified current collector. Interestingly, we apply this concept to fabricate the first prototype of 18,650-type cylindrical aqueous-based supercapacitors, correlating to the size and shape of commercial devices.

## Results and discussion

### Morphologies and physical properties

**Figure 1:**
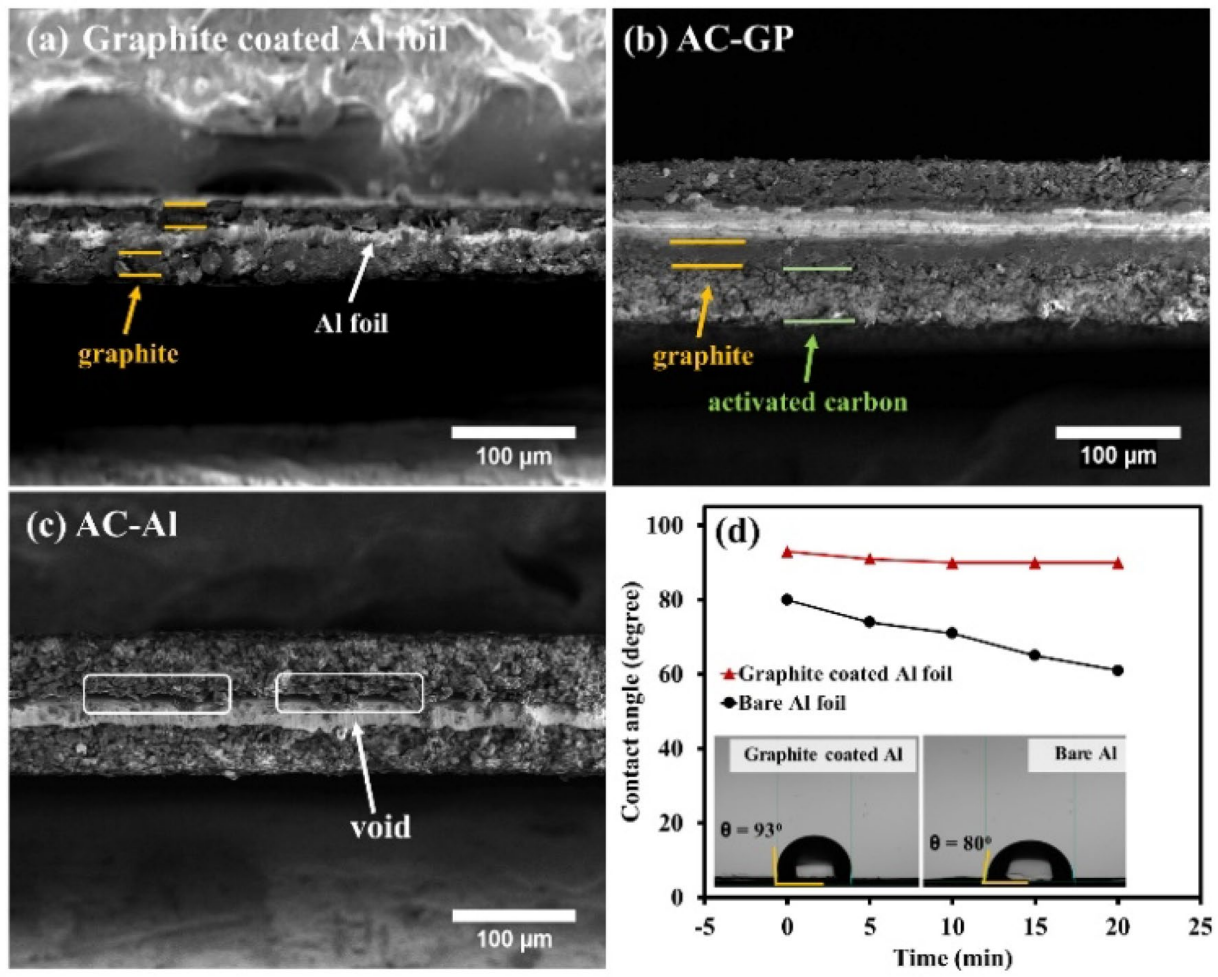
FESEM images of (**a**) graphite-coated Al foil, (**b**) AC-GP, and (**c**) AC-Al; (**d**) contact angle vs. immersion time (inset: the drops of 1 M Na_2_SO_4_ at t = 0 min).

Graphite slurry was firstly coated on the top and bottom of Al foil surface as a protective layer. The activated carbon as an active material was subsequently coated on the graphite-coated Al foil current collector (so-called “AC-GP”) and bare Al foil (so-called “AC-Al”). Note, the specific surface area of activated carbon was measured by BET method which is ~ 2159 m^2^ g^−1^ (see Fig. S4a and Table S1). In Fig. 1a, the cross-sectional SEM image shows that the graphite was successfully coated on Al surface with a thickness of *ca.* 50 µm together with the Al current collector. Note, the weight of current collector before and after graphite coating are 4.1 and 5.3 mg cm^−2^, respectively. The AC-GP shows a strong interfacial contact between the active material layer and the as-modified current collector without air void space (see Fig. 1b), while the AC-Al exhibits void space between the material layer and the current collector (see Fig. 1c). This is due to a strong interfacial contact between carbon materials (graphite and activated carbon) as a nature of materials. Importantly, only activated carbon coated on Al foil exhibits void space while graphite shows a strong adhesion without the gap. The reason is from the different morphologies of these materials as shown in Fig. S5. It can be seen that the graphite shows more plate-like morphology as compared to activated carbon. Then, after graphite coating on Al foil, graphite arranged in a parallel-plate configuration with layer-by-layer to each other and parallel to Al foil, resulting in ordered packing and high adhesion between graphite layer and Al foil. While, activated carbon exhibits 3-D architectures with various shapes which might lead to unordered packing so the layer of activated carbon can be detached easily from Al foil.

Apart from morphologies, a time-dependence wettability of the as-modified substrate was also analysed using the Ossila Contact Angle in a high humidity chamber using a fixed droplet volume of 10 μL of 1 M Na_2_SO_4_ electrolyte as shown in Fig. 1d. The graphite-coated Al surface exhibits a hydrophobic property with a contact angle (CA) of 93° at t = 0^19^. Then, it is slightly reduced to 90° at t = 20 min. In contrast, the bare Al foil demonstrates explicitly hydrophilic behaviour with a CA value of 80° at t = 0 and reducing to 61° at t = 20 min. This is due to the hydrophilic property of the native oxide (Al_2_O_3_) thin film on the surface of Al foil^20^. It is clear that the graphite results in an increasing CA of the substrate, indicating that water is prohibited to penetrate to reach the Al foil surface.

### Electrochemical evaluation

**Figure 2:**
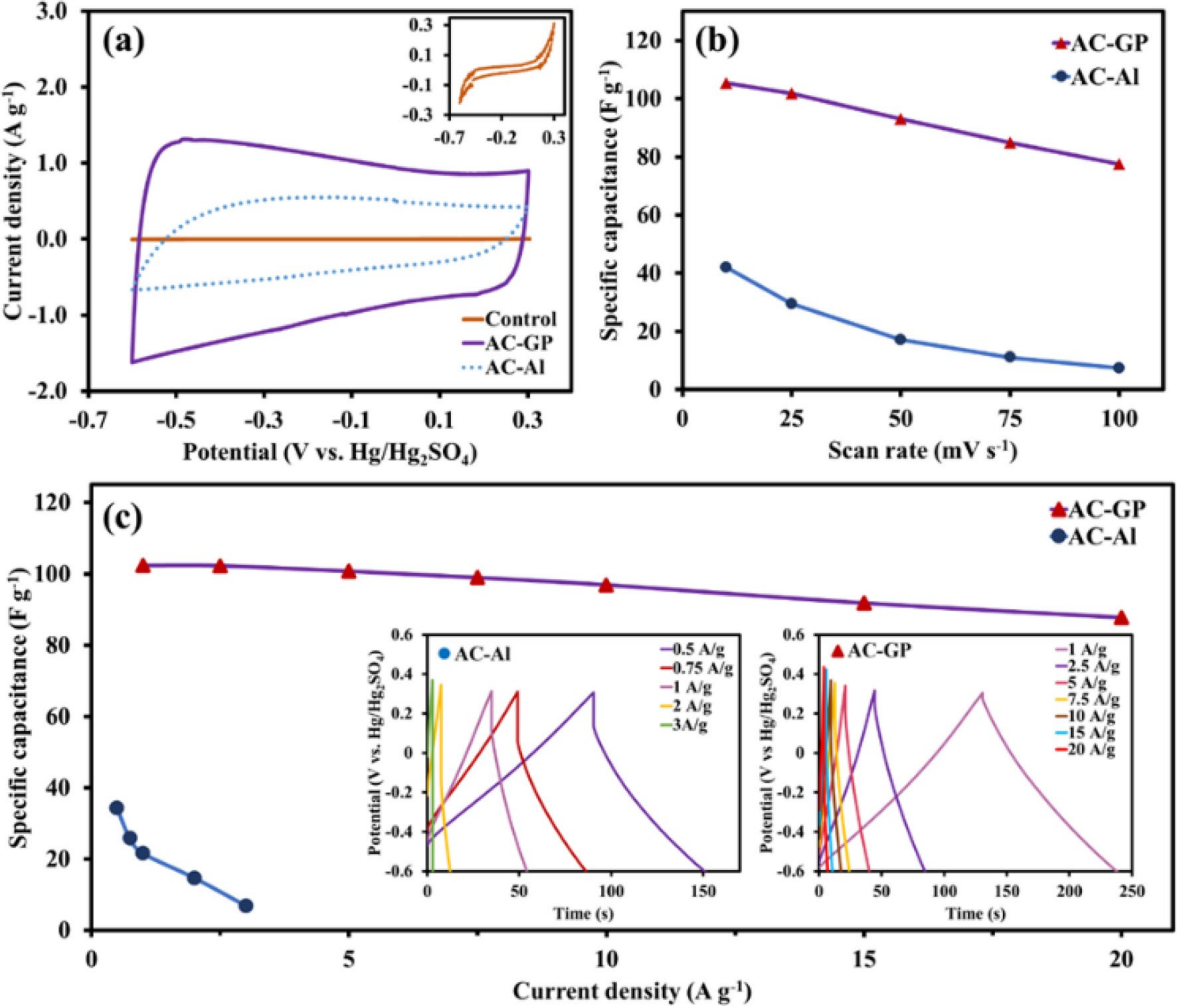
(**a**) CVs at 10 mV s^−1^ (inset: CV profile of a control); and (**b**) specific capacitances at different scan rates; and (**c**) specific capacitances as a function of current densities (inset: GCD profiles at various current densities).

To confirm that the interfacial contact is involved in the electrochemical performance of the as-prepared electrodes, the CV and GCD were performed in a three-electrode system at a potential range from − 0.6 to 0.3 V *vs*. Hg/Hg_2_SO_4_ using 1 M Na_2_SO_4_ as the electrolyte. The comparison of the CV profiles between the as-prepared electrodes with and without graphite layer at 10 mV s^−1^ (Fig. 2a) demonstrates that the current collector with the graphite layer shows a more rectangular CV shape and a larger integrated area under the CV curve, corresponding to lower internal resistance and higher specific capacitance^21^. As expected, the AC-GP exhibits a higher specific capacitance (105.3 F g^−1^) than the AC-Al (42.1 F g^−1^) at 10 mV s^−1^. Moreover, the CV profile of AC-GP retains an almost rectangular shape with a capacitance of 77.6 F g^−1^ and a retention of 74% even at an increased scan rate of 100 mV s^−1^ (see Figs. 2b and S6a). This indicates a high level of electronic conductivity from good interfacial contact between the electrode material and the graphite-coated current collector^22^. Meanwhile, the AC-Al shows a distortion of the CV curves (see Fig. S6c), indicating high internal resistance due to the formation of resistive oxide film and poor interfacial contact^21^. The reason was further explained in the *ex-situ* investigation section. Additionally, the AC-Al shows rapid capacitance fading, maintaining 18% at 100 mV s^−1^ (Fig. 2b) due to the reasons discussed above. In addition, the electrochemical performance of graphite-coated Al foil was evaluated as a control that it provides only 1.06 mF g^−1^ at 10 mV s^−1^ which is negligible (see inset of Fig. 2a). This is due to its extremely low surface area of 8 m^2^/g as obtained from BET measurement (see Fig. S4b and Table S1).

Apart from the CV, the electrochemical performance was also investigated using the GCD with respect to applied current densities. Figure 2c shows that the AC-Al exhibits a smaller specific capacitance when compared with that of the AC-GP at the same current density. We can ascribe this to the formation of oxides on the Al foil surface during cycling, leading to reduced capacitance^23^. The insets in Fig. 2c show the GCD profiles of AC-GP and AC-Al for which the GCD profiles of AC-GP exhibit a more symmetrical triangular shape in which a coulombic efficiency of the AC-GP at 1 A g^−1^ is 84.2% that is higher than 47.8% of the AC-Al, indicating better electrochemical reversibility^24^. In addition, the AC-GP shows a much smaller iR drop (25 mV) when compared to the AC-Al (199 mV) at 1 A g^−1^, in which the iR drop corresponds to the internal resistance, indicating that the AC-Al has a higher resistance because of the formation of more Al_2_O_3_ resistive film on the Al foil surface^25^. This internal resistance leads to poor rate capability as shown in Fig. 2c, for which the AC-Al performs well only at low current densities (from 0.5 to 3 A g^−1^), with a rapid fading in capacitance at 80% (from 29.2 to 6.8 F g^−1^). Conversely, the AC-GP shows outstanding rate capability with a capacitance fading only 14% even at 20 A g^−1^ (87.7 F g^−1^) as compared to 1 A g^−1^ (102.4 F g^−1^), showing a strong capability for high-rate charge/discharge properties.

**Figure 3:**
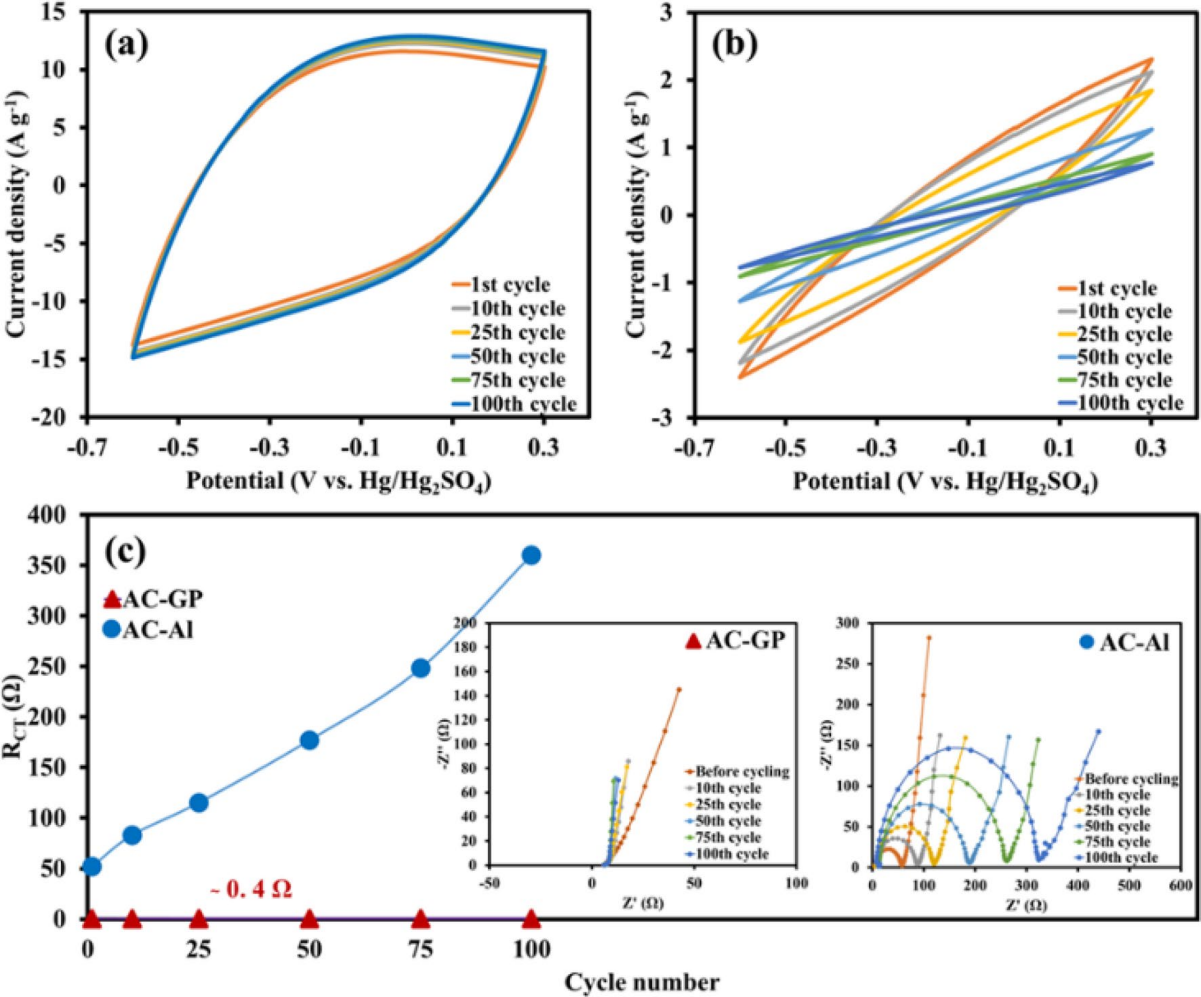
CVs at 100 mV s^−1^ of (**a**) AC-GP and (**b**) AC-Al and (**c**) Summarized R_CT_ values as a function of cycle numbers (inset: Nyquist plots).

To further evaluate the passive oxide film on the Al foil, CV coupled with electrochemical impedance spectroscopy (EIS) was carried out on two different samples with and without the graphite coated layer. The results are shown in Fig. 3. The samples were subjected to 100 cycles at a scan rate of 100 mV s^−1^ and the EIS was then applied before cycling and after the 10th, 25th, 50th, 75th, and 100th cycles. The CV profiles of the AC-GP maintain an almost rectangular shape even after the 100th cycle (Fig. 3a), while the AC-Al shows a higher distortion with increased CV cycles (Fig. 3b), suggesting an increasing internal resistance from the resistive oxide film grown on the Al foil during long cycling^21^. This can be more clearly observed from the Nyquist plots of the samples after cycling. The EIS was evaluated at a voltage amplitude of 10 mV over the frequency range from 50 kHz to 0.01 Hz at an open-circuit potential (*OCP*) as shown in Fig. 3c. Overall, the charge transfer resistance (*R*_*CT*_) is assigned to the contact resistance at the interface between the electrode material and the current collector, which can be evaluated from the semicircle in the Nyquist plot (inset Fig. 3c). The plot in Fig. 3c shows that the AC-GP electrode exhibits almost the same charge transfer resistance (about 0.4 Ω) after 100 cycles, which suggests that the good interfacial contact of AC-GP remains unchanged after long cycling. In contrast, the *R*_*CT*_ of AC-Al increases from 52.2 to 360.2 Ω after cycling because of the formation of a resistive oxide layer (Al_2_O_3_) as well as an increase in void spaces at the interface of the electrode after long cycling^26,27^.

**Figure 4:**
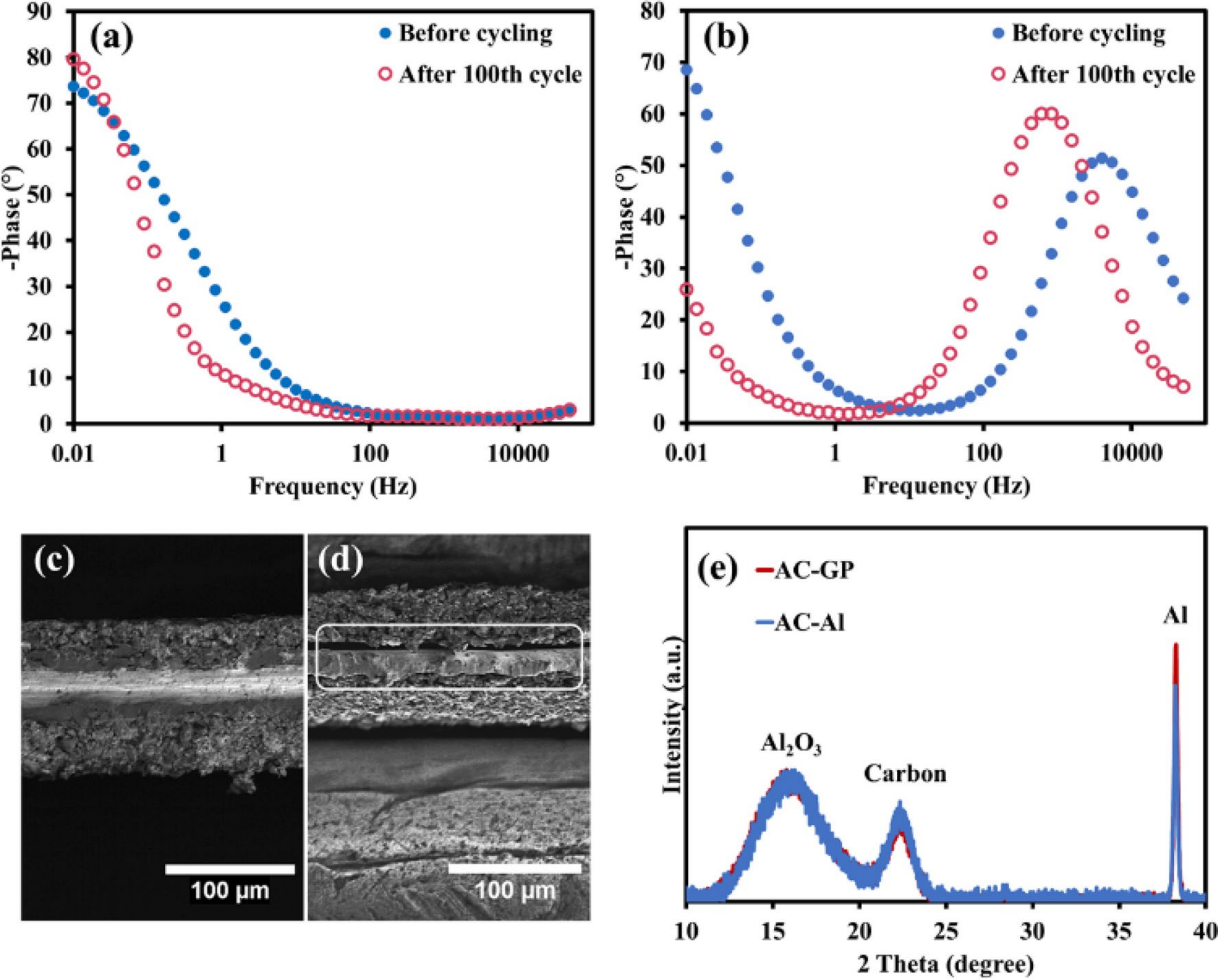
Bode phase diagrams of (**a**) AC-GP and (**b**) AC-Al and ex-situ cross-sectional SEM images of (**c**) AC-GP and (**d**) AC-Al images after the 100th cycle via CV, and (**e**) ex-situ XRD patterns of Al_2_O_3_ thin film compared with Al of the as-prepared electrodes after cycling for 100 cycles.

In addition to the Nyquist plot, Bode phase diagrams were analysed (see Fig. 4a,b). It has been found that the electrode with a phase angle close to -90° represents an ideal capacitor behavior^28^. The AC-GP phase angles are closer to -90° (Fig. 4a) when compared to the AC-Al (Fig. 4b), signifying the improved capacitive behaviour. Moreover, the Bode diagrams of AC-GP and AC-Al show different profiles. While the AC-GP profiles display only EDLC behaviour, the AC-Al profiles show the characteristic peak of pseudocapacitive behaviour because of the formation of the passive oxide film on the Al foil^1^. In addition, the intensity of this peak increases after 100 cycles, indicating an increasing oxide film formation during cycling. The formation of the oxide film leads to poor capacitive behaviour, as indicated by the change of phase angles of AC-Al from − 68.6° to − 25.9° after long cycling.

The passive oxide film and the void space were further investigated by the ex-situ characterization. The ex-situ SEM was carried out to confirm the void space generated after 100 cycles, which can clearly be observed in the cross-sectional images. The AC-GP shows an excellent level of the interfacial contact between the activated carbon layer and the graphite-modified Al foil even after 100 cycles (Fig. 4c), resulting in an excellent rate capability and a high level of stability. In contrast, the AC-Al exhibits a more detached active material layer from the Al surface (Fig. 4d) since the oxide film on the Al surface reduces the adhesion between the AC active material and the Al current collector^29^.

Moreover, the generated oxide film on the Al surface after long cycling can be further confirmed by the Al_2_O_3_ thin film as analysed by the ex-situ XRD (see Fig. 4e). The ratio between Al_2_O_3_ and Al can be obtained from the XRD using the relative intensities of Al_2_O_3_ peak at 2θ = 15.9° (Al_2_O_3_ phase)^30^ and Al peak at 2θ = 38.2° (111 plane)^31^. The AC-Al shows a higher Al_2_O_3_ content of about 14.6% as compared with that of the AC-GP, indicating the graphite layer can reduce the Al_2_O_3_ film formation. Note, the Al_2_O_3_ film spontaneously grows on the Al foil surface at atmospheric conditions having humidity. The characteristic peak of carbon shows a typical wide-angle XRD pattern at 22° indicating the 002 plane of carbon, which represents the carbon materials^32^.

**Figure 5:**
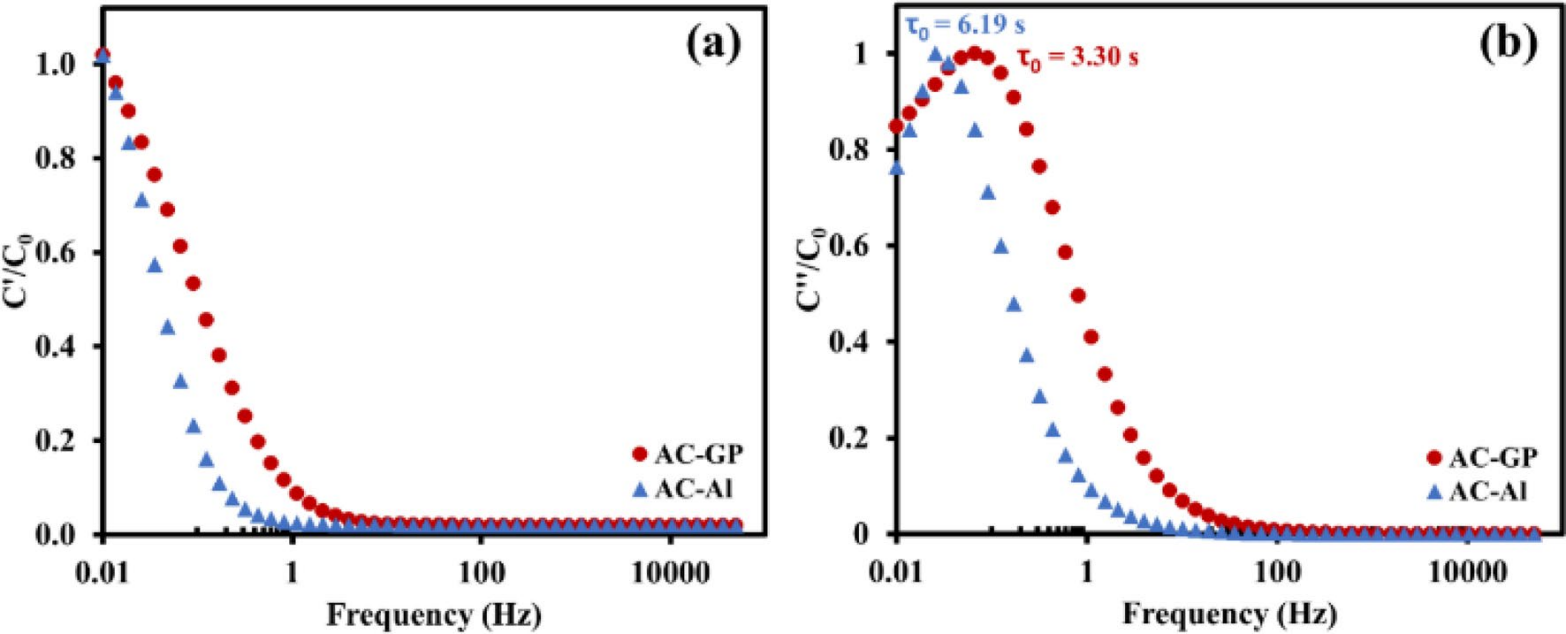
(**a**) real and (**b**) imaginary parts of the normalized capacitances as a function of frequencies.

To further study the capacitive properties of those two samples, a complex capacitance model was applied according to Eqn. S3–S4. In Fig. 5a, the plot between the normalized real-part capacitances and frequencies refers to the capacitive and resistive behaviours of the electrodes. At high-frequency region, it shows a resistor behaviour due to the fast polarization while the capacitive behaviour presents at lower frequencies due to a slower polarization, thus electrolytic ions have enough time to diffuse through the electrode. The transition region from the resistive behaviour to capacitive behaviour of AC-GP is shifted to a higher frequency as compared with AC-Al, meaning that the system of AC-GP is able to react to a faster changing polarization due to its higher conductivity^33^.

Moreover, the kinetics of the electrode can be further described using the relaxation time constant (*τ*_*0*_) in Bode plots of imaginary capacitances, where τ_0_ refers to the minimum time required to discharge stored energy, which can be obtained from the peak frequency(*f*_0_, τ_0_ = 1/*f*_0_) (see Fig. 5b)^34^. The τ_0_ values of AC-GP and AC-Al are 3.30 and 6.19 s, respectively, indicating a faster charge/discharge capability for the AC-GP, which represents a higher rate of both capability and power density.

**Figure 6:**
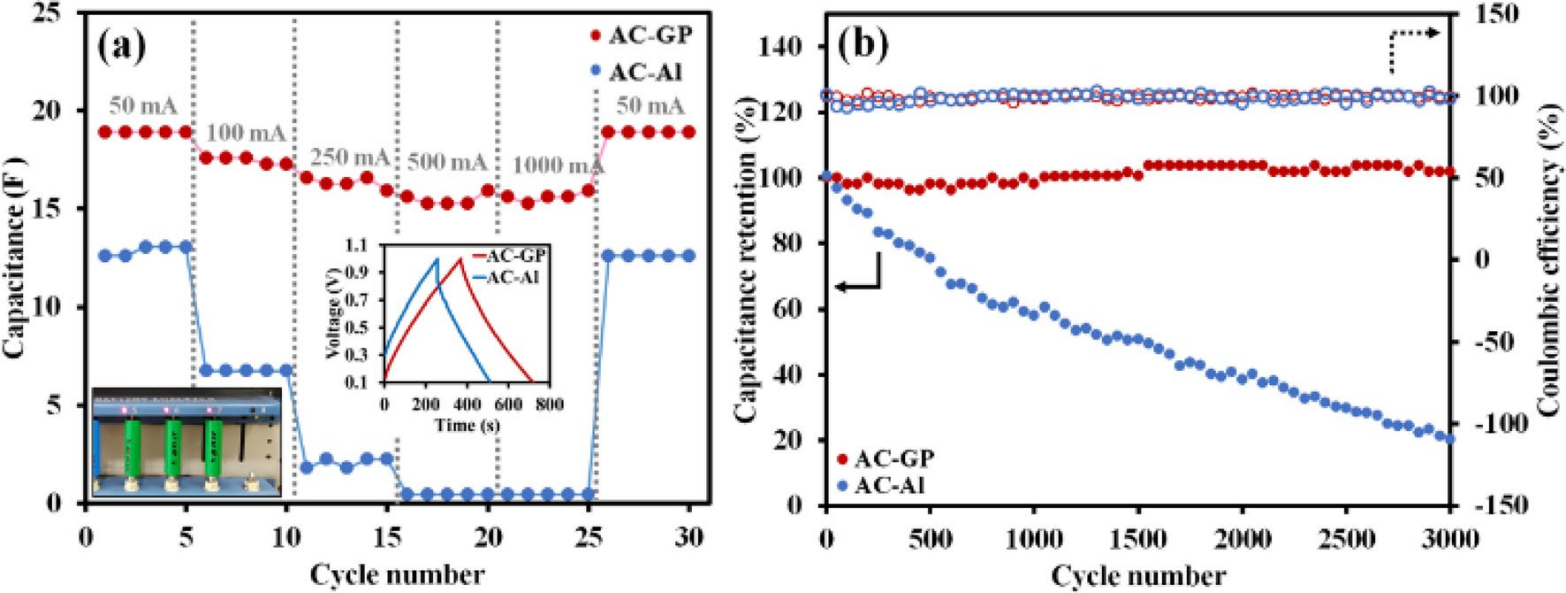
(**a**) Rate capability of the 18,650 cylindrical cells of AC-GP and AC-Al, and (**b**) stability at 100 mA.

Finally, we have successfully fabricated the first prototype of 18,650-type supercapacitors in an aqueous electrolyte system using 1 M Na_2_SO_4_. The charge storage performance was investigated using the GCD method from 50 to 1000 mA as shown in Figs. 6a and S7. As expected, the AC-GP in Fig. 6a shows a higher cell capacitance than the AC-Al. Additionally, as compared to GCD profiles at 50 mA, the AC-GP exhibits a lower *iR* drop (13 mV) than that of AC-Al (85.6 mV) (see inset of Fig. 6a), indicating a better electrical conductivity in the electrode^35^. Moreover, the AC-GP electrode shows an excellent maintenance rate capability with remained at 82.4% even at 1000 mA. Unlike the AC-GP, the AC-Al can perform at currents of less than 250 mA and the capacitance retention is reduced 17.2% when the current is increased from 50 to 250 mA. Moreover, the stability of the assembled devices was further evaluated at 100 mA. Figure 6b shows that the AC-GP is maintained at almost 100% of capacitance after 3000 cycles. In contrast, the AC-Al exhibits a rapid capacitance fade, and only 19.4% of its capacitance is maintained because of the corrosion of the Al foil (the formation of oxides) as well as an increase in void space over long cycling. Moreover, the AC-GP was further evaluated cycling performance at a higher current of 250 mA in which AC-GP performs without capacitance fading even at 10,000th cycle, indicating good cycling stability (see Fig. S8). Additionally, the assembled devices were further investigated in terms of their internal resistance by following the IEC 62391-1 standard (Fig. S9). The internal resistance was described in terms of the equivalent series resistance (ESR), and the ESR of AC-GP shows a smaller resistance (0.2 Ω) than the AC-Al (0.6 Ω) which leads to a higher conductivity rate in the electrode, resulting in a higher power delivery of the device.

**Figure 7:**
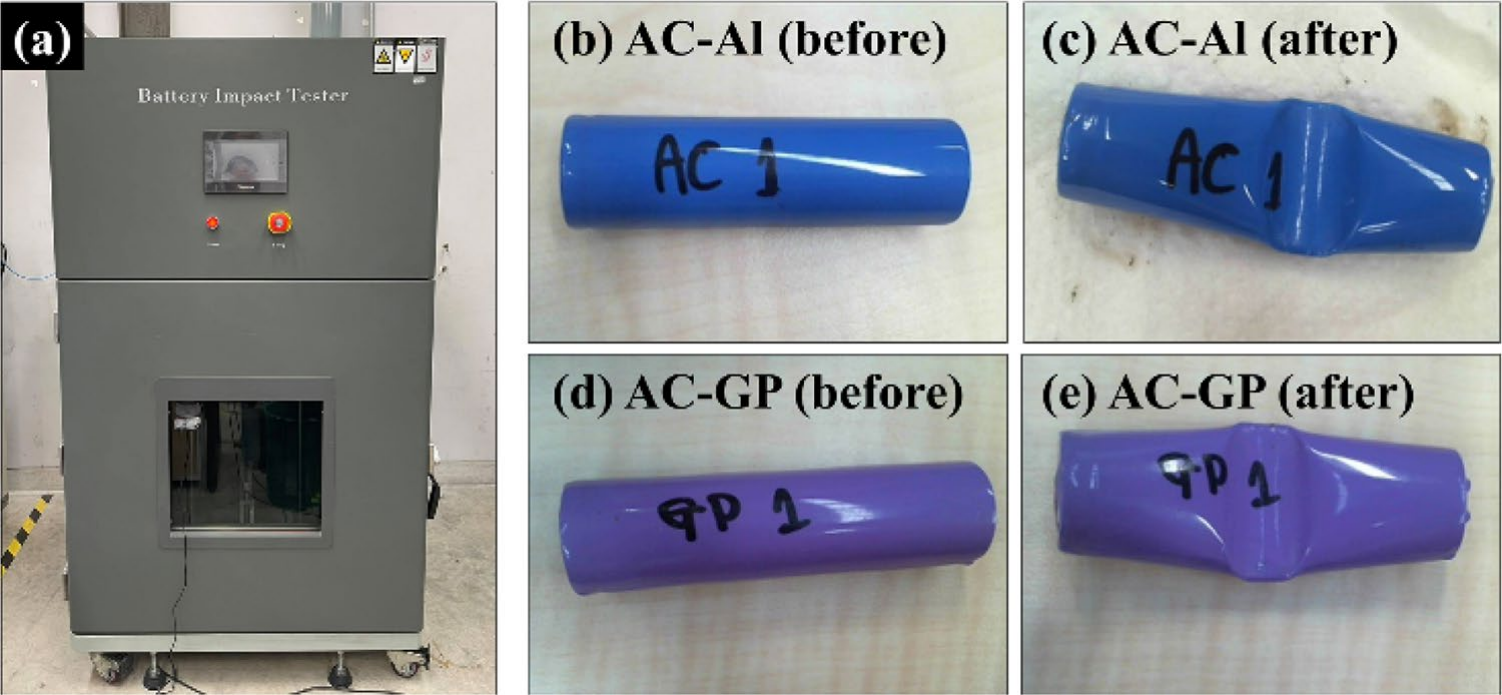
(**a**) Battery impact tester, (**b**, **c**) AC-Al cylindrical cell, and (**d**–**e**) AC-GP cylindrical cells before and after the impact test according to the UN38.3 standard.

In addition, the as-fabricated cylindrical supercapacitors were evaluated via impact test (UN38.3) to confirm the safety of the cylindrical cells during use in real applications such as in electric vehicles. For example, if a car has been in an accident by a crash, various abuse modes will occur such as thermal, mechanical, and electrical modes which might lead to a dangerous incident. Thus, this test can be used to confirm the safety of the cylindrical cell that needs to have no disassembly and no fire of the cell after a crash. The impact tester machine used for the test was shown in Fig. 7a. The cylindrical supercapacitors with 1 M Na_2_SO_4_ aqueous electrolyte of both AC-Al and AC-GP samples were fully charged before testing. For the impact test process, a stainless steel 316 bar of 9.2 kg at the height of 610 cm will drop and crash the cylindrical cell. All the as-fabricated aqueous cylindrical cells exhibit no explosion during impact test, indicating safe energy storage devices for e-mobility applications as shown in Fig. 7b–e and videos in the supporting information. It should be noted that the shadow as we can observe in the video is not the flame but it is light reflected on a water-based electrolyte when the electrolyte was leaking out of the case after the crash.

## Conclusions

The anticorrosion graphite layer was successfully coated on the Al foil current collector by the semi-automation roll-to-roll coating process at the production pilot plant of 18,650 cylindrical cells. The graphite layer can improve the interfacial contact between the activated carbon active material layer and the Al foil current collector enhancing the charge transfer or reducing the internal resistance of the cells. Also, it helps to preserve the surface of the Al foil current collector, preventing the formation of inactive insulating oxide film (Al_2_O_3_). The electrode with the graphite layer (AC-GP) demonstrates a higher specific capacitance (105.3 F g^−1^) than that of AC-Al (42.1 F g^−1^) at 10 mV s^−1^ as well as better stability in which the AC-GP shows excellent cycling stability over 10,000 cycles. This work then may lead to practical large-scale supercapacitors for future sustainable energy storage applications.

## Experimental section

### Chemicals and materials

Graphite (particle size of < 20 μm, Sigma-Aldrich), Activated carbon (TF-B520, MTI), Carbon black (Super P, TIMCAL), *N*-methyl pyrrolidinone (99.5% 1-Methyl-2-Pyrrolidone, QRec), Polyvinylidene fluoride (PVDF, Mw ∼534,000, Sigma-Aldrich), Sodium sulphate (Na_2_SO_4_, CARLO ERBA) were used without any purification. Deionised water was purified using Milli-Q system (DI water, 15 MΩ cm, Millipore). Aluminium foil (thickness of 18 µm, GELON LIB) was used as a current collector.

### Fabrication of supercapacitor electrodes

First, the Al foil was modified by coating the graphite as a protective layer onto both the top and bottom of Al foil surface by the roll-to-roll coating technology (see Figs. S1 and S2). The graphite slurry was prepared by mixing graphite with conductive carbon black (Super P) and polyvinylidene fluoride (PVDF) binder in a finely tuned weight ratio of 83:7:10 in N-methyl pyrrolidinone (NMP). The finely tuned total solid content is about 20% and then kept stirred for 10 h to obtain a homogenous slurry. The as-prepared graphite slurry was coated onto the surface of Al foil using a roll-to-roll coating machine as shown in Fig. S1a in a dry room with a dew point of − 40 °C. Then, the graphite coated Al foil was dried at 120 °C under vacuum for 24 h before coating an active material. Then, the activated carbon slurry was prepared by the following graphite slurry preparation using activated carbon instead of graphite but the finely tuned solid content of activated carbon slurry is about 15%. The homogeneous activated carbon slurry was coated onto the top and bottom of a graphite coated aluminium foil as demonstrated in Fig. S1b. In addition, the activated carbon was also coated onto Al foil without graphite coating to evaluate the effect of the graphite passivation layer (see Fig. S2). Finally, all the electrodes were dried at 120 °C under vacuum for 48 h. Note, all the electrodes were pressed at a finely tuned 1 ton before electrochemical measurements and the active mass loading of all the as-prepared electrodes is about 3.5 mg cm^−2^.

### Physicochemical characterizations

Textural properties of the activated carbon and graphite powder were studied by N_2_ adsorption/desorption measurement at 77 K (BELSORP-mini, Microtrac BEL Crop). Prior to measurement, all the samples were degassed at 373 K for 48 h. The specific surface area was determined by Brunauer-Emmette Teller (BET) model. The total pore volume was calculated from the amount of N_2_ adsorbed at a relative pressure (*P*/*P*_*0*_) of 0.95. The pore diameter was calculated from *D*_*P*_ = 4*V*_*total*_/*S*_*BET*_. Furthermore, the cross-sectional morphology of the as-prepared electrodes was investigated by Field-emission scanning electron microscopy using the beam energy of 1.0 keV (FESEM, JSM7001F, JEOL Ltd.). The crystalline oxide products grown on Al foil surface have been identified by X-ray diffraction (XRD) patterns from powder X-ray diffraction (PXRD, Bruker D8 ADVANCE) using Cu-Kα radiation (λ = 1.5418 Å, 40 kV, 40 mA) with a step size of 0.01° within the 2θ region of 10–40°. The identification of the XRD patterns was identified using the JCPDS data (JCPDS 11-0517)^30^. In addition, contact angle tests were carried out to identify the property of the current collector surfaces with and without graphite coating layer in which 1 M Na_2_SO_4_, the electrolyte used in this work, was dropped (10 µL) on the surface of current collectors. The droplet images were taken by the Ossila Contact Angle Goniometer.

### Electrochemical measurements

The electrochemical evaluation of the electrode was first carried out in a three-electrode configuration in 50 mL of 1 M Na_2_SO_4_ electrolyte. The as-prepared electrodes were firstly cut into 1 cm^2^ and were then used as a working electrode. The polycrystalline platinum and the mercury/mercury sulphate (Hg/Hg_2_SO_4_) electrode were used as a counter electrode and a reference electrode, respectively. The electrochemical performance of the as-prepared electrodes was investigated by cyclic voltammetry (CV) and galvanostatic charge/discharge (GCD) with a recording between − 0.6 and 0.3 V vs. Hg/Hg_2_SO_4_. The specific capacitance from CV and GCD can be obtained from the Eqs. (S1) and (S2), respectively (see the supporting information). Note, the specific capacitance was calculated by using the mass of active material which is only activated carbon. The EIS was carried out in a frequency range from 50 kHz to 0.01 Hz at an open circuit potential with a voltage amplitude of 10 mV. Note, prior to electrochemical measurement, the as-prepared electrodes were activated via CV at a scan rate of 10 mV s^−1^ for 20 cycles. The 18,650 cylindrical cells were then investigated rate capability and stability via the GCD method by using a battery tester (Neware, Gelon, Hong Kong).

## Supplementary Information

Supplementary Information 1.

Supplementary Video 1.

Supplementary Video 2.
