# Supplementary material for: Scalable 18,650 aqueous-based supercapacitors using hydrophobicity concept of anti-corrosion graphite passivation layer
Source: Sci Rep. 2021 Jun 22;11:13082. doi: 10.1038/s41598-021-92597-y (PMC8219742; doi:10.1038/s41598-021-92597-y)
Supplement: Supplementary file 1 — Supplementary Information 1. [file 41598_2021_92597_MOESM1_ESM.docx]

**Supporting information**

**Scalable 18650 Aqueous-based Supercapacitors Using Hydrophobicity Concept of Anti-corrosion Graphite Passivation Layer**

Praeploy Chomkhuntod,^a^ Pawin Iamprasertkun,^b^ Poramane Chiochan,^a^ Phansiri Suktha,^a^ and Montree Sawangphruk*,^a^

^a^Centre of Excellence for Energy Storage Technology (CEST), Department of Chemical and Biomolecular Engineering, School of Energy Science and Engineering, Vidyasirimedhi Institute of Science and Technology, Rayong 21210, Thailand.

^b^Department of Applied Physics, Faculty of Sciences and Liberal Arts, Rajamangala University of Technology Isan, Nakhon Ratchasima, 30000, Thailand.

**EXPERIMENTAL SECTION**

Contact angle measurement using the aqueous electrolyte

*Graphite-coated aluminium foil*


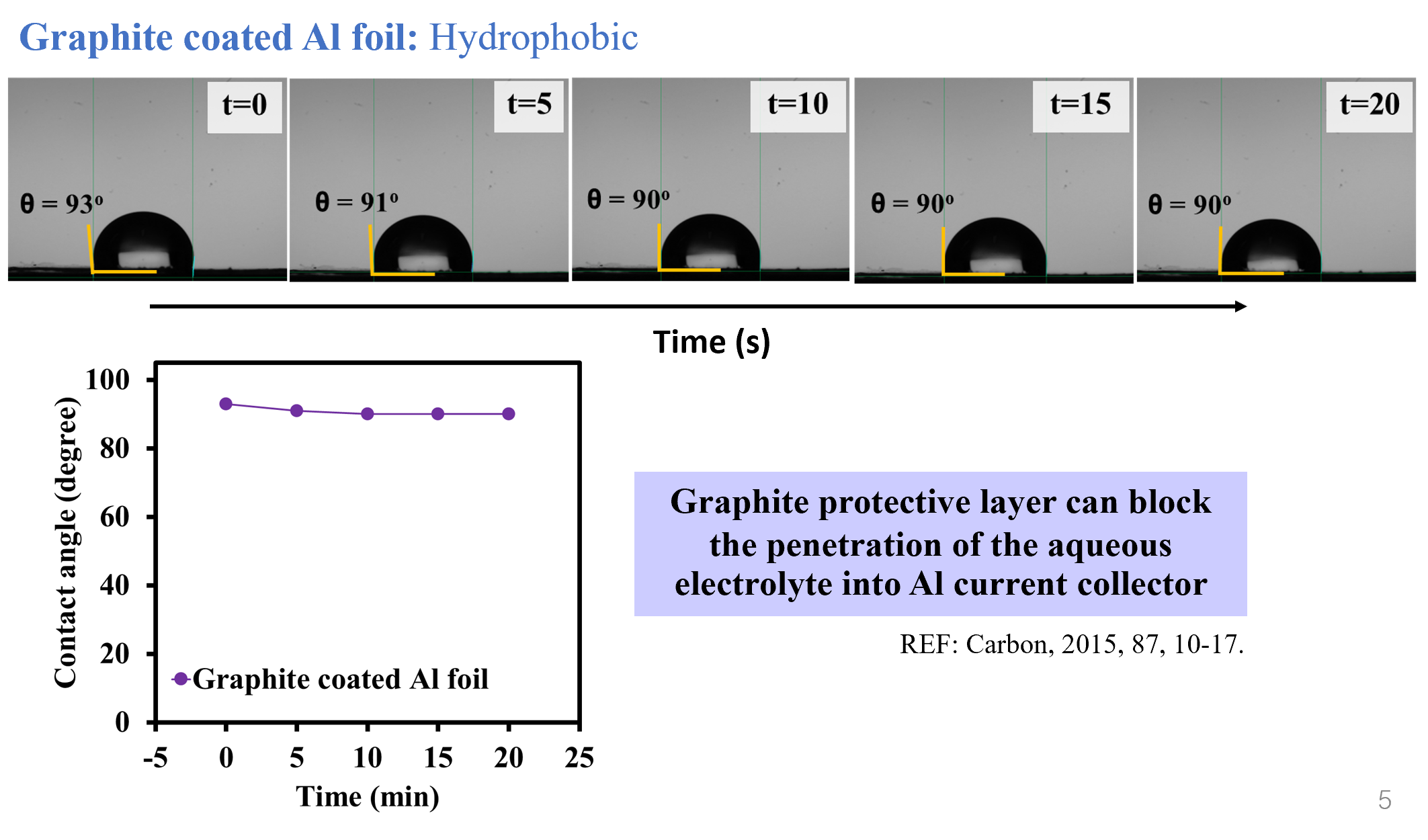


*Al foil (control)*


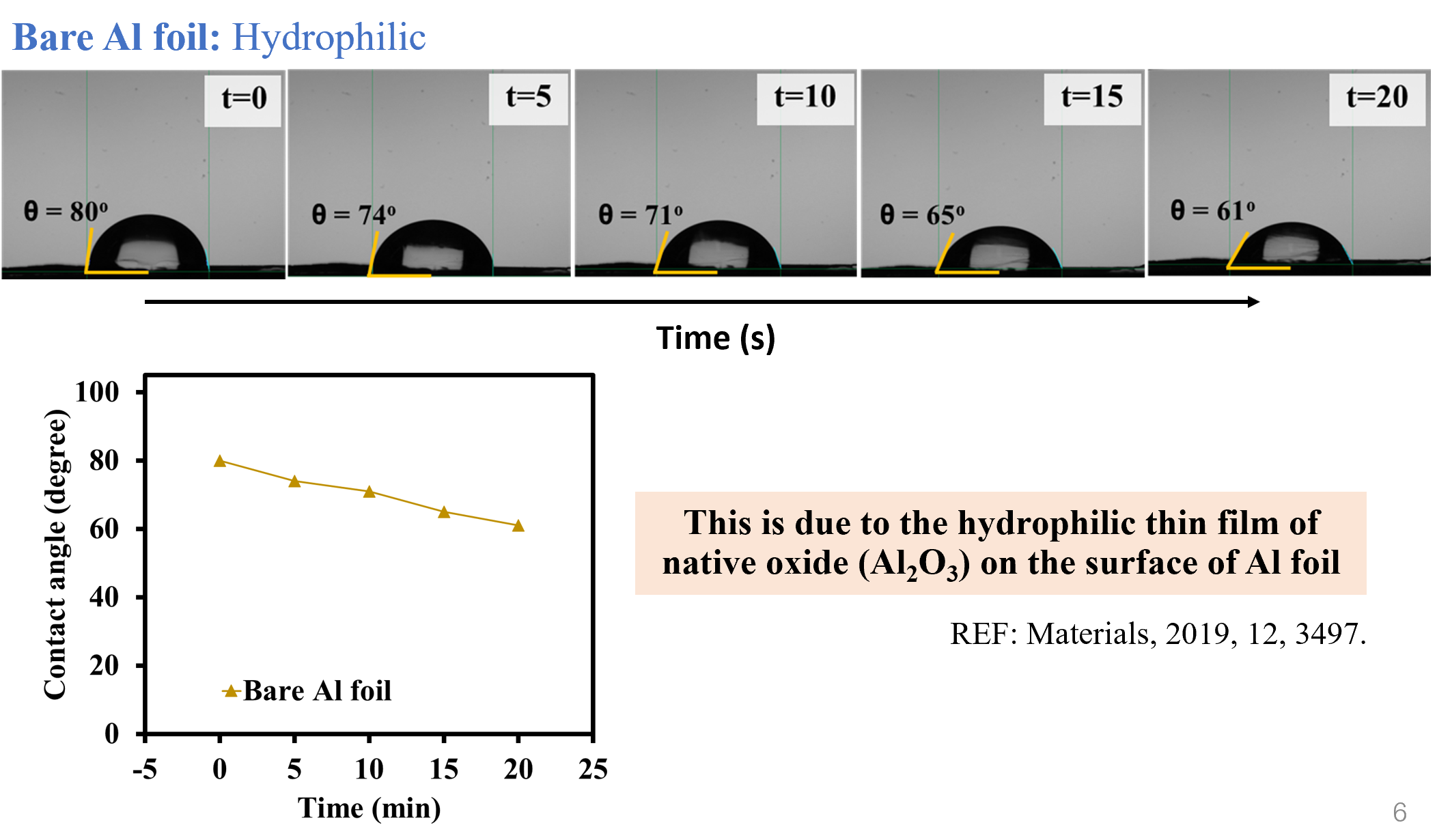


**Electrode preparation**

The current collector was modified by coating the graphite as a protective layer on top and bottom of aluminium (Al) foil surface. Firstly, the graphite slurry was prepared by mixing graphite with conductive carbon black (Super P) and polyvinylidene fluoride (PVDF) binder in a weight ratio of 83:7:10 in N-methyl pyrrolidinone (NMP). The total solid content is about 20% and then kept stirred for 10 h to obtain a homogenous slurry. The as-prepared graphite slurry was coated onto the surface of Al foil using a roll-to-roll coating machine as shown in Fig S1a. Secondly, the as-modified current collector was dried at 120 ^°^C under vacuum for 24 h before coating an active material, activated carbon. Then, the activated carbon slurry was prepared by following the same slurry preparation as the graphite (using activated carbon instead of graphite) but the solid content of activated carbon slurry is about 15%. The homogeneous activated carbon slurry was coated onto the top and bottom surfaces of Al foil by using a roll-to-roll coating machine as demonstrated in Fig S1b. Then, the electrode was dried at 120 °C under vacuum for 48 h. In addition to the modified electrode, the activated carbon was coated onto Al foil (without graphite coating) for comparing the electrochemical performances of the electrodes (Fig. S2). Note, all the as-prepared electrodes were pressed at 1 ton before the electrochemical measurement and the active mass loading of all the as-prepared electrodes is about 3.5 mg cm^-2^.


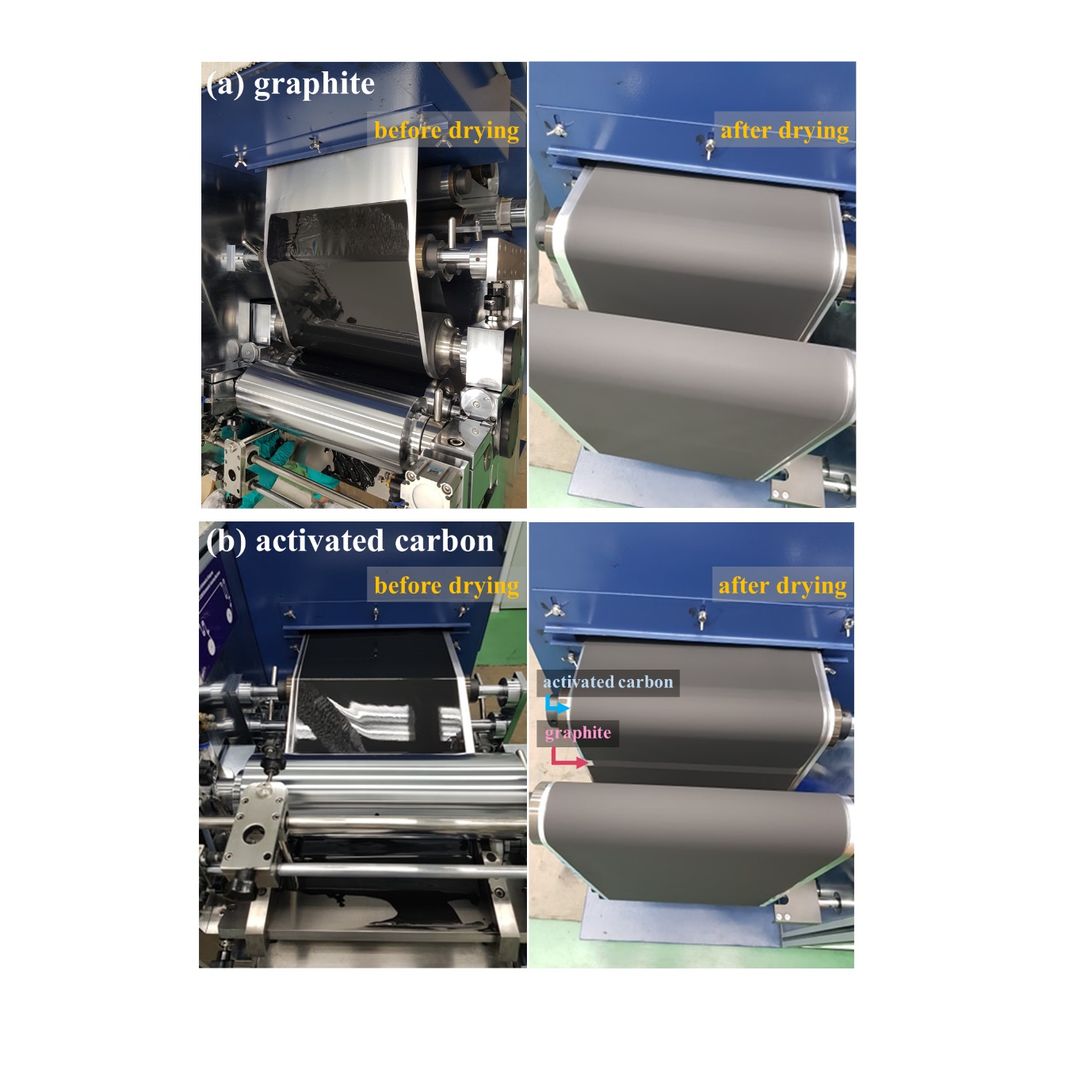


**Figure S1**. Electrode preparation using a roll-to-roll coating machine of (a) graphite on bare aluminium foil and (b) activated carbon coated on graphite coated aluminium foil.


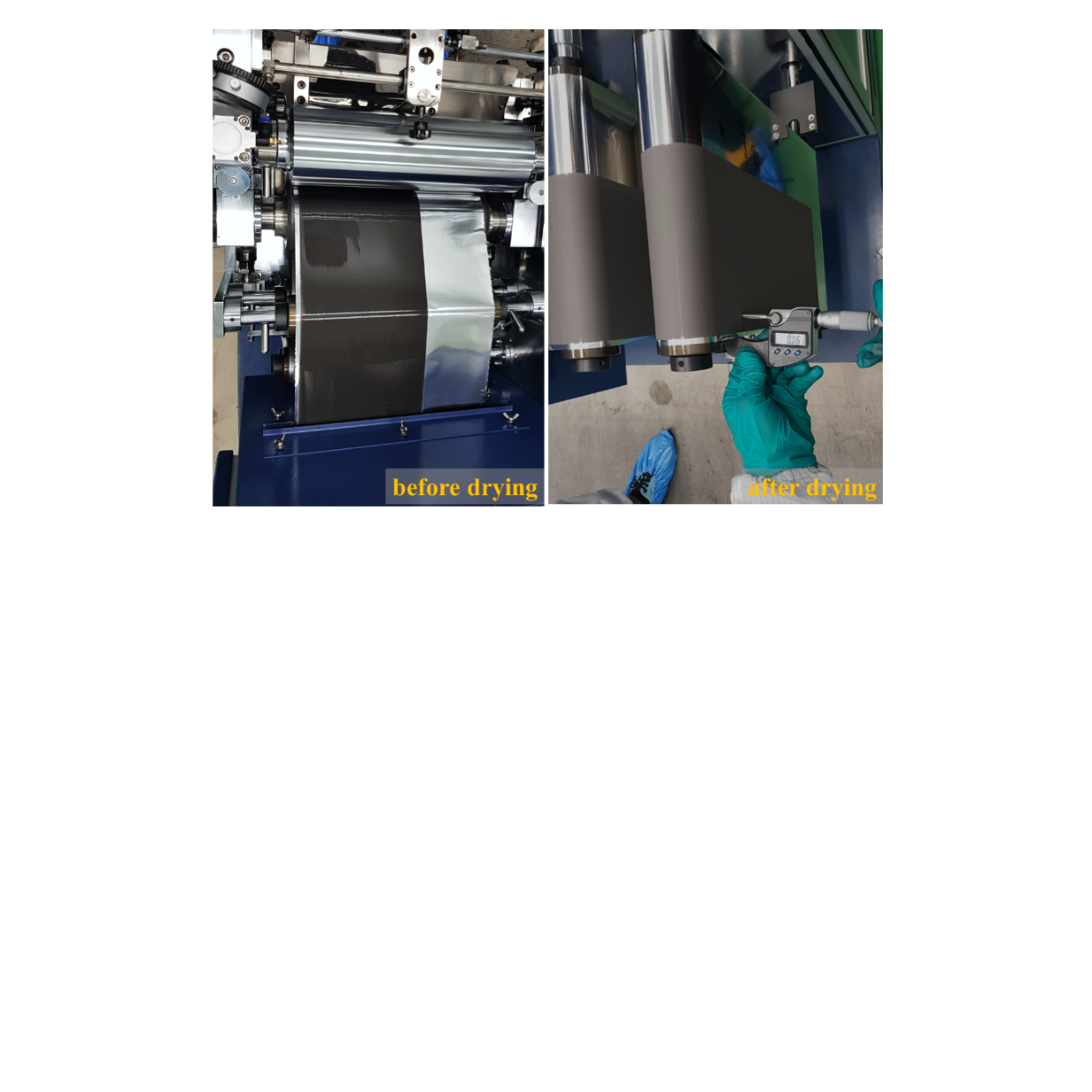


**Figure S2**. Electrode preparation of activated carbon coated on bare aluminium foil using a roll-to-roll coating machine.

**Cell Assembly**

The 18650 cylindrical cell fabrication was demonstrated briefly in Fig. S3. Firstly, the as-prepared electrodes were slit and cut into the size of 26. 3 cm (Length) x 5.6 cm (Width) as demonstrated in Fig. S3a. The hydrolysed polyethylene (PE) with a thickness of 175 µm was used as a separator with a size of 70 cm (L) x 5.8 cm (W). Then, the two symmetric electrodes and a separator were rolled together by using a winding machine in which the as-rolled electrodes were shown in Fig. S3b. Then, the as-rolled electrodes were put into the case and weld with the case and the cap (Fig. S3c). Finally, each cell was injected with 1 M Na_2_SO_4_ electrolyte fully (*ca.* 5 g of the electrolyte). Prior to measurement, all the as-assembled cylindrical cells were aged for 2 days.

**
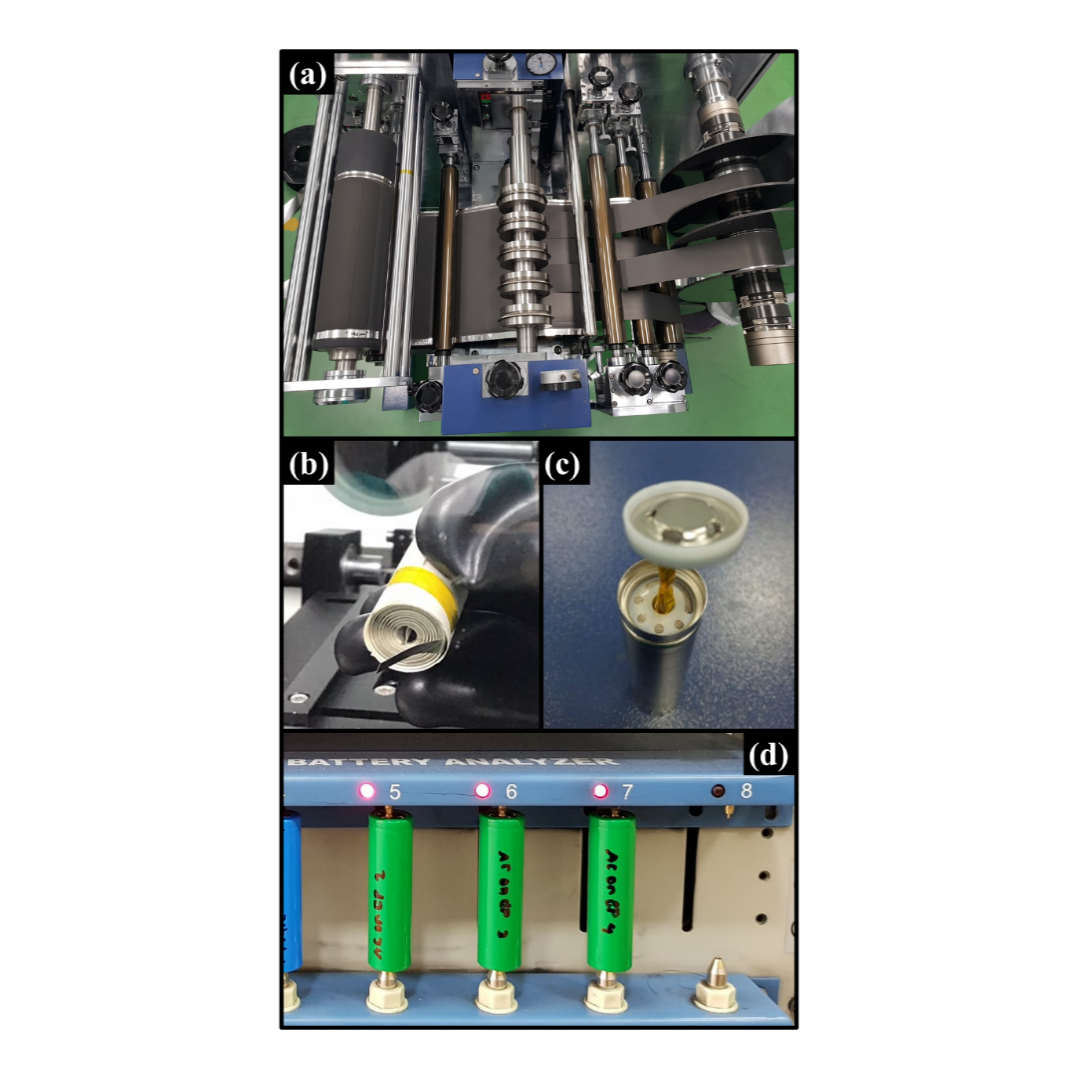
**

**Figure S3**. 18650 cylindrical cell fabrication; (a) slitting of electrode, (b) the as-winded electrodes coupled with a separator, and (c) the as-welded electrodes with the case.

**The electrochemical calculation**

**For three-electrode configuration**

The specific capacitance of the prepared samples can be calculated from both CV and GCD following equation (S1) and (S2)^1-3^;

**For CV**

$C_{CV}= \int\frac{IdV/v}{m\Delta V}$ (S1)

where *I* is the response current, ∫𝐼𝑑𝑉/𝑣 (Coulomb) is the integrated area of the CV (discharge process) which refers to the total amount of charge on the surface of the electrode, ∆𝑉 is the potential window, and *m* is the mass loading of active material.

**For GCD**

$C_{GCD}=\frac{I\Delta t}{\Delta V}$ (S2)

where *I* is the applied current density, ∆𝑉 is the potential window of discharge, Δ*t* is the discharge time.

To further analyse the impedance data, the real and imaginary parts of the complex capacitance were then calculated by the following equation (S3) and (S4), respectively.^2,4^

*𝐶*'(*𝜔*) = ‒ *𝑍*"(*𝜔*)/{*𝜔*|*𝑍*(*𝜔*)|^2^} (S3)

*𝐶*"(*𝜔*) = *𝑍*'(*𝜔*)/{*𝜔*|*𝑍*(*𝜔*)|^2^ (S4)

where *𝜔 =* 2*𝜋𝑓* (angular frequency), *Zʹ* and *Z"* represent the real and imaginary parts of the complex impedance *Z*, respectively.

**Relaxation time constant (*τ_0_*)**

The *τ_0_* can be obtained from the reciprocal of the peak frequency (*𝑓_0_*) in the plot between normalized capacitance (*C*″(*ɷ*)) and frequency according to the equation below (S5);^5^

*𝜏_0_ =* 1/(2*𝜋𝑓_0_)* (S5)

**For supercapacitor device**

The capacity (mAh) of as-assembled 18650 cylindrical cell was obtained from the battery tester, then it was conversed to the capacitance (F) by following equation below (S6);^6^

$F=\frac{mAh}{dV\left( V \right)}\cdot\frac{1 A}{1000 \mathrm{mA}}\cdot\frac{3600 s}{1 h}=\frac{i(A)\cdot dt(s)}{dV\left( V \right)}$ (S6)

where *F* is total cell capacitance, *i* is an applied current, *t* is discharge time, and ∆𝑉 is the potential window of discharge.

**Equivalent series resistance (ESR) and equivalent distributed resistance (EDR)**

The as-fabricated devices were further evaluated the resistance *via* GCD method according to the IEC 62391-1 standard. The ESR was calculated from the *iR* drop at the beginning of the discharge at 100 mA after holding at 1 V for 30 min as well as the EDR was calculated from the discharge profile (see Fig. S9), using the equations below;^7^

$ESR=\frac{\Delta V_{ESR}}{\left| I_{discharge} \right|}$ (S7)

$EDR=\frac{\Delta V_{EDR}}{\left| I_{discharge} \right|}$ (S8)

**Characterizations**

Textural properties of the activated carbon and graphite powder were studied by N_2_ adsorption/desorption measurement at 77K (BELSORP-mini, MicrotracBEL Crop). Prior to measurement, all the samples were degassed at 373 K for 48 h. The specific surface area was calculated by Brunauer-EmmetteTeller (BET) model. The total pore volume was calculated from the amount of N_2_ adsorbed at a relative pressure (*P*/*P_0_*) of 0.95. The pore diameter was calculated from *D_P_* = 4*V_total_*/*S_BET_***.** Furthermore, the as-prepared electrode

The cross-sectional morphology of the as-prepared electrodes was investigated by Field-emission scanning electron microscopy using the beam energy of 1.0 keV (FESEM, JSM7001F, JEOL Ltd.).

The crystalline oxide products grown on Al foil surface have been identified by X-ray diffraction (XRD) patterns from powder X-ray diffraction (PXRD, Bruker D8 ADVANCE) using CuKα radiation (λ = 1.5418 Å, 40 kV, 40 mA) with a step size of 0.01° within the 2θ region of 10-40°. The identification of the XRD patterns was identified using the JCPDS data (JCPDS 11-0517).^8^

Contact angle tests were carried out to identify the properties of the current collector surfaces in a comparison between with and without graphite coating layer in which 1M Na_2_SO_4_ was dropped (10 µL) on the surface of current collectors. The droplet images were taken by the Ossila con angle machine.

**
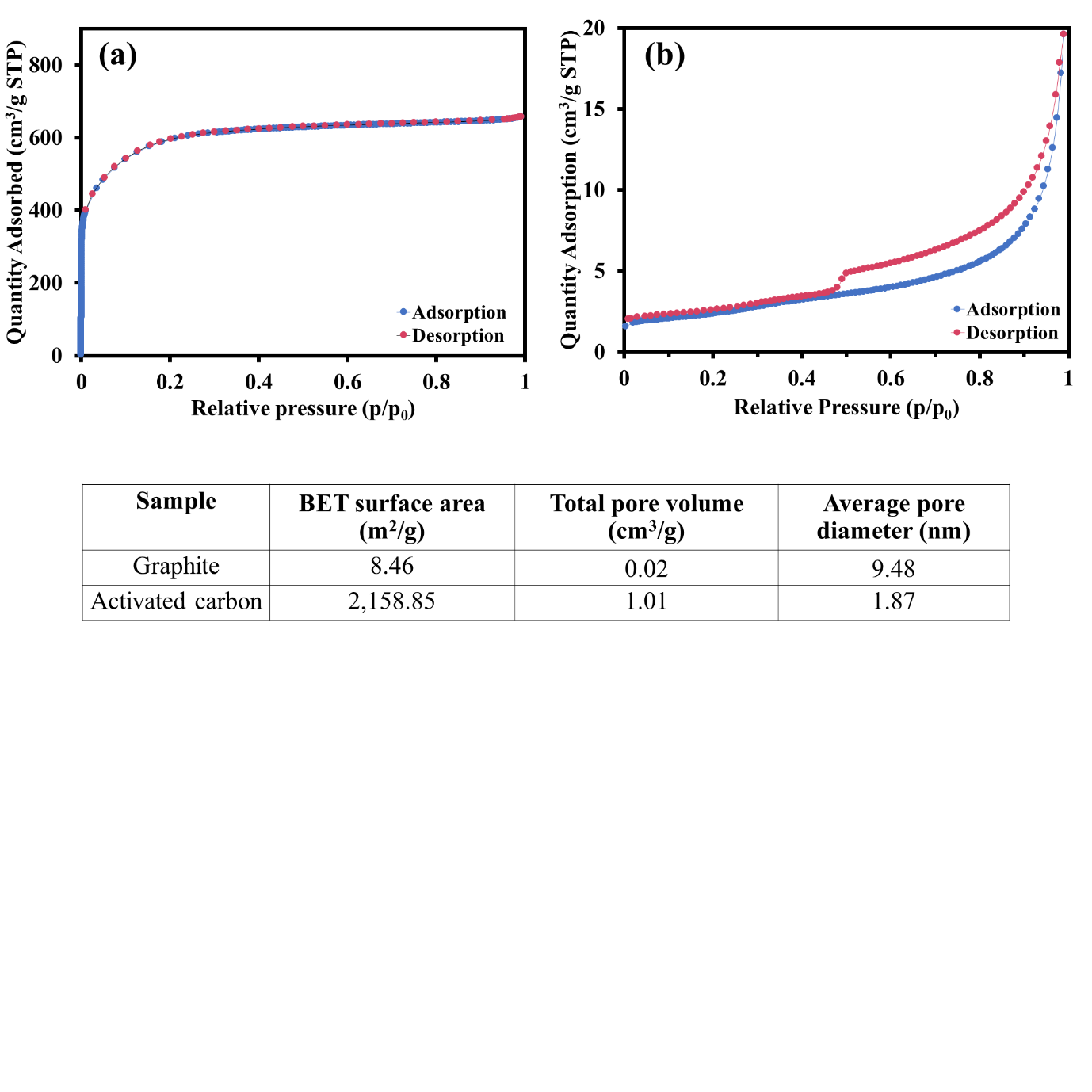
**

**Figure S4.** N_2_ adsorption/desorption isotherms of (a) activated carbon and (b) graphite powder**.**

**Table S1.** N_2_ adsorption/desorption data

| **Sample** | **BET surface area (****m^2^/g)** | **Total BJH pore volume (cm^3^/g)** | **Average BJH pore diameter (nm)** |
| --- | --- | --- | --- |
| Activated carbon | 2,159 | 1.01 | 1.87 |
| Graphite | 8 | 0.02 | 9.48 |

**
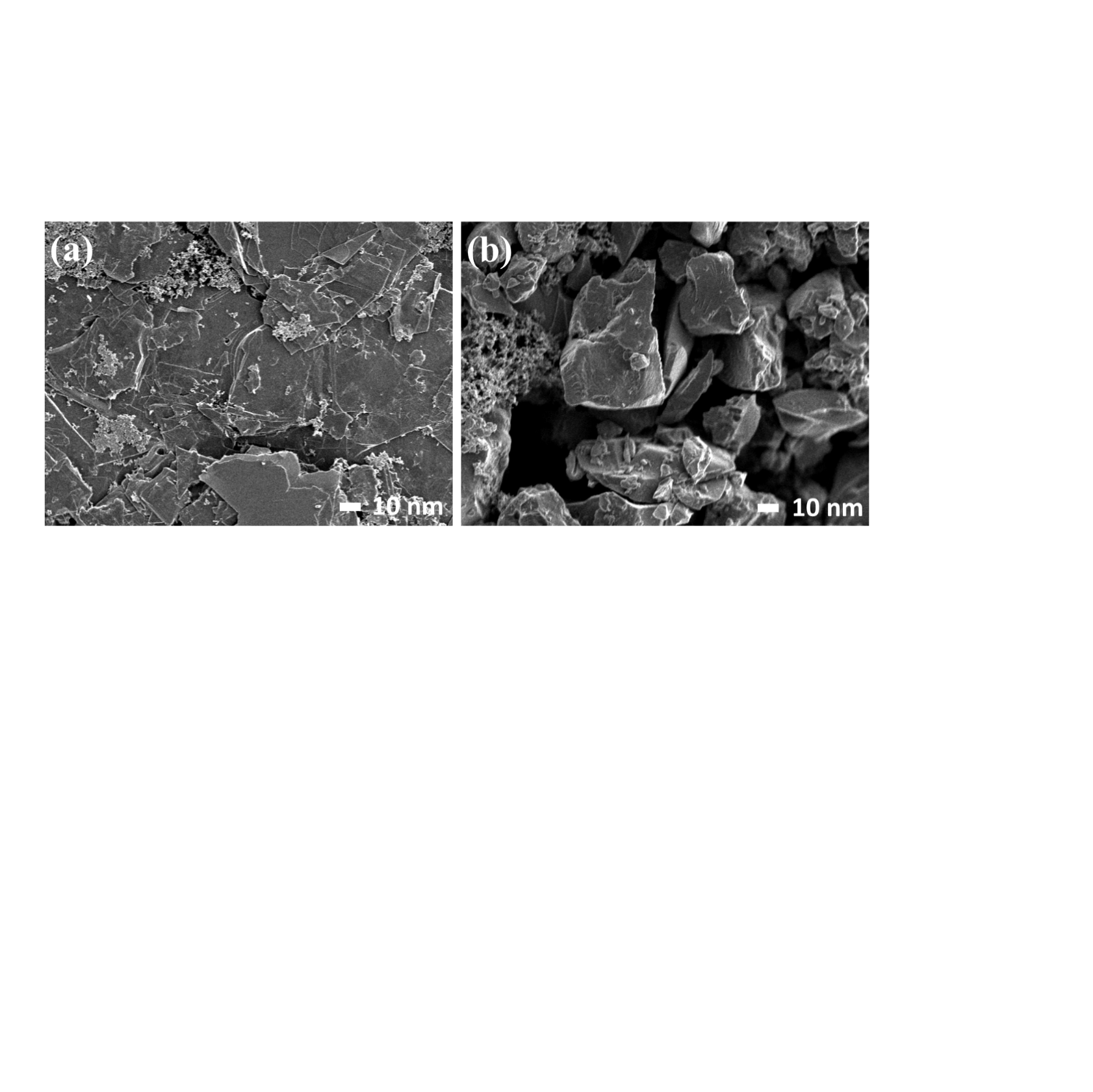
**

**Figure S5.** Top-view SEM images of (a) graphite and (b) activated carbon coated on Al foil.

**
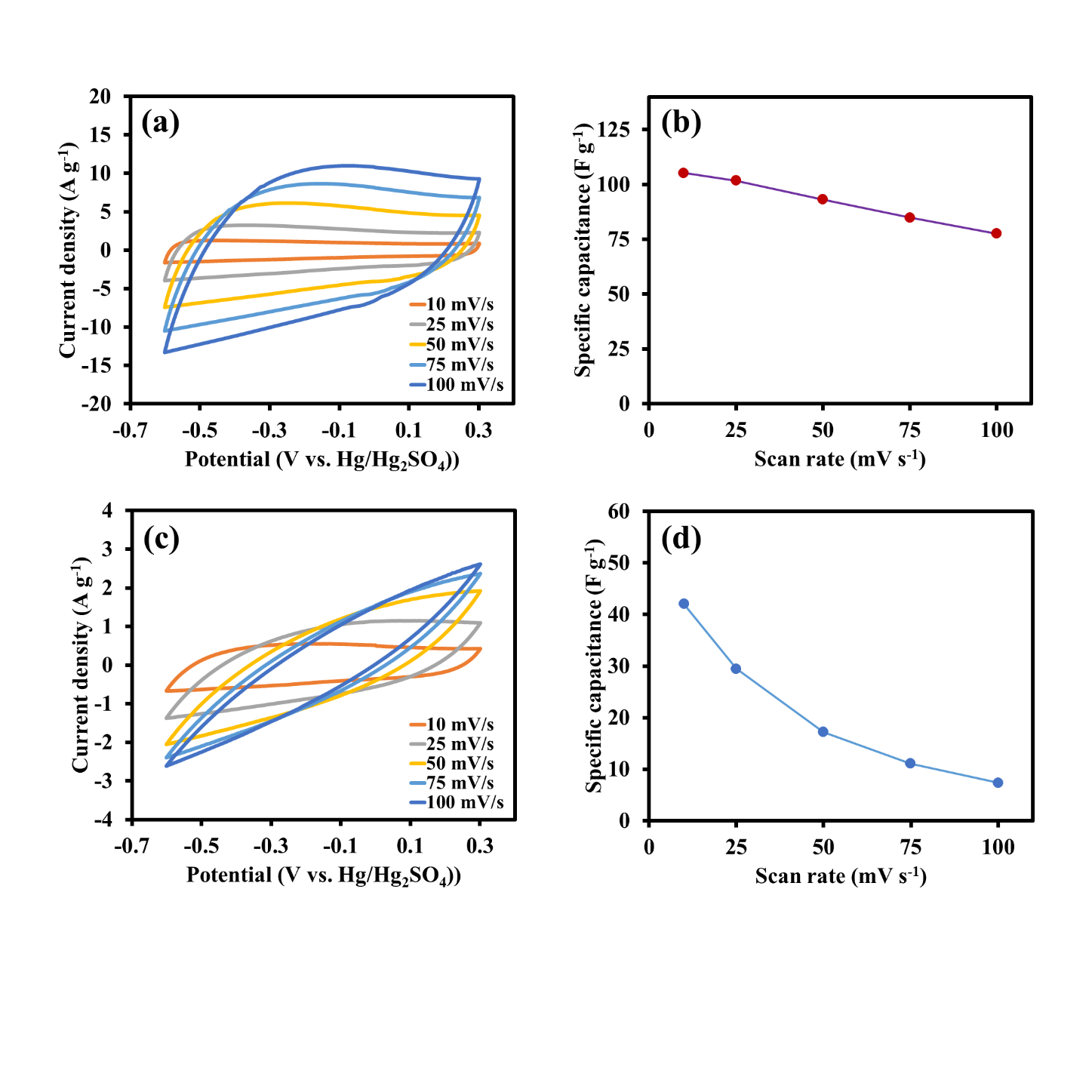
**

**Figure S6.** CV curves and specific capacitances as a function of scan rates of (a-b) AC-GP and (c-d) AC-Al.

The as-prepared electrodes were further investigated at different scan rates from 10 to 100 mV s^-1^. The AC-GP exhibits a remained nearly rectangular shape of CV profile even at a scan rate of 100 mV s^-1^ (Fig. S6a), maintaining 74% capacitance retention (Fig. S6b), in which the specific capacitances of AC-GP are 105.3, 101.8, 93.1, 84.9, and 77.6 F g^-1^ at scan rates of 10, 25, 50 ,75, and 100 mV s^-1^, respectively. On the other hand, the AC-Al shows a significant distortion of CV profiles during an increase of scan rate (Fig. S6c) and maintains only 18% of the capacitance retention (Fig. S6d) in which specific capacitances are 42.1, 29.5, 17.2, 11.1, and 7.3 F g^-1^ at scan rates of 10, 25, 50,75, and 100 mV s^-1^, respectively. This due to that the AC-Al possesses higher internal resistance as compared to AC-GP as a result of the resistive oxide layer and void space at the interface between the electrode material layer and current collector.


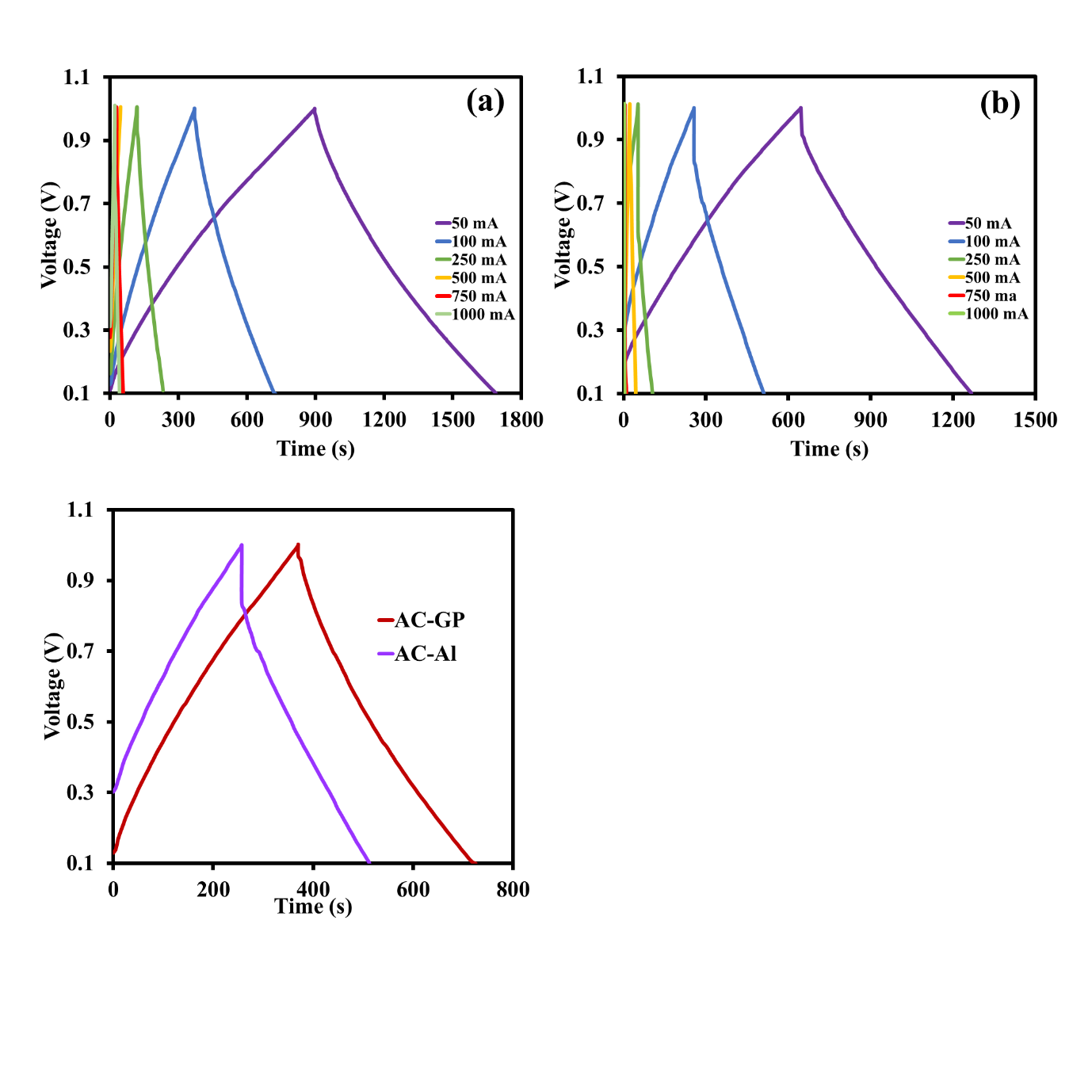


**Figure S7.** GCD curves at different current densities of symmetric cylindrical cells of (a) AC-GP and (b) AC-Al.


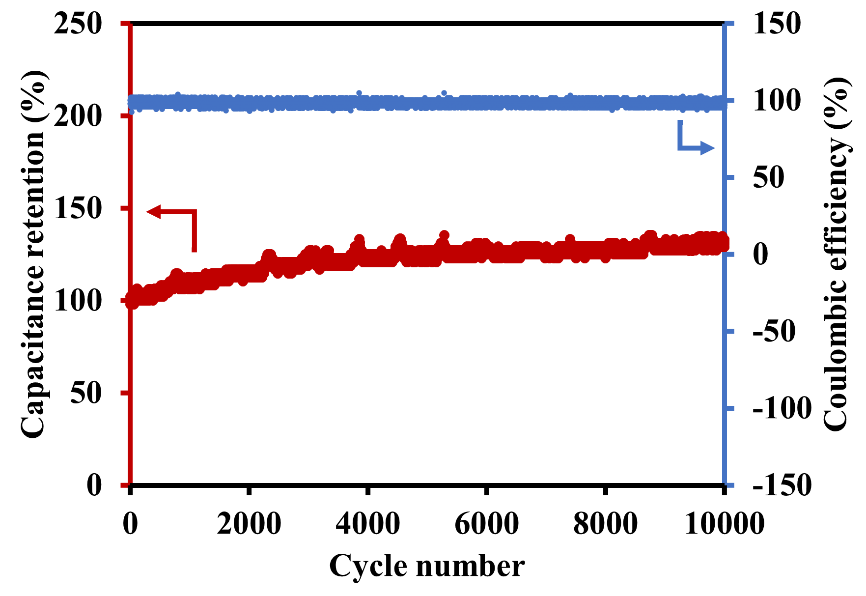


**Figure S8.** Stability testing of AC-GP at a current of 250 mA.


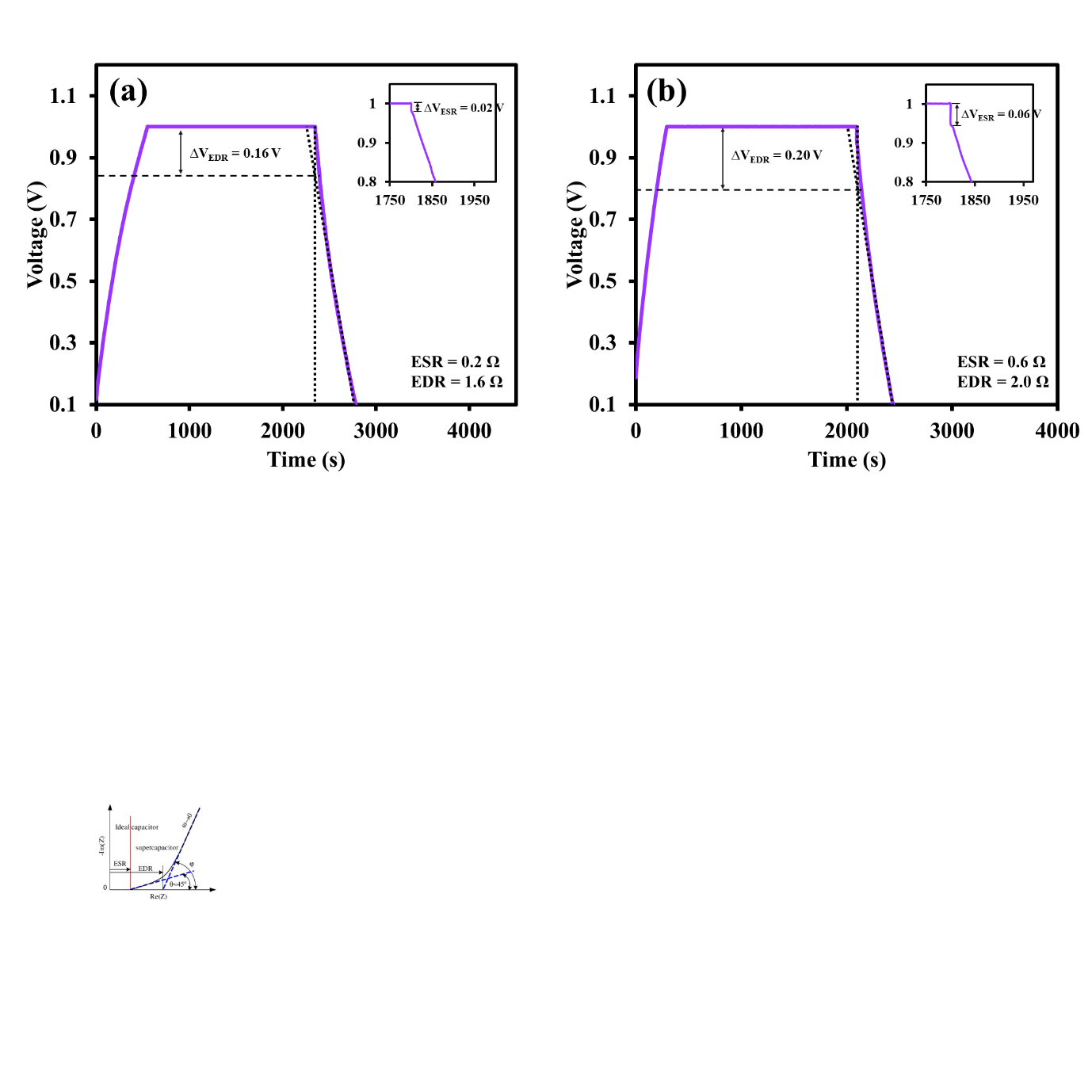


**Figure S9.** Charging and discharging voltage curve at a dwelling time of 30 min of (a) AC-GP and (b) AC-Al used for EDR and ESR evaluation.

The equivalent series resistance (ESR) represents the sum of the resistances of the device including the bulk electrolyte, the resistance of the electrode, as well as the contact resistance between the electrode and the current collector. However, some supercapacitors exhibit the equivalent distributed resistance (EDR) which is the ESR plus an ionic resistance from the electrolyte for the charge redistribution process within the pores of the electrode.^7^ The ESR and EDR were calculated by following the equations (S7) and (S8). The AC-GP shows smaller ESR (0.2 Ω) than AC-Al (0.6 Ω). Besides, the EDR values of AC-GP and AC-Al are 1.6 and 2.0 Ω, respectively, indicating that the same resistance from the ions within the porous material (1.4 Ω) due to the same electrode material (activated carbon).

**Supporting References**

1 Meng, F.-L. *et al.* Integrated Cu3N porous nanowire array electrode for high-performance supercapacitors. *J. Mater. Chem. A* **5**, 18972-18976, doi:10.1039/C7TA05439D (2017).

2 Ma, N. *et al.* Effect of intercalated alkali ions in layered manganese oxide nanosheets as neutral electrochemical capacitors. *Chem. Commun.* **55**, 1213-1216, doi:10.1039/C8CC08198K (2019).

3 Zhu, S. *et al.* Structural Directed Growth of Ultrathin Parallel Birnessite on β-MnO2 for High-Performance Asymmetric Supercapacitors. *ACS Nano* **12**, 1033-1042, doi:10.1021/acsnano.7b03431 (2018).

4 Lee, K. *et al.* Highly transparent and flexible supercapacitors using graphene-graphene quantum dots chelate. *Nano Energy* **26**, 746-754, doi:<https://doi.org/10.1016/j.nanoen.2016.06.030> (2016).

5 Bhattacharjya, D. *et al.* Fast and controllable reduction of graphene oxide by low-cost CO2 laser for supercapacitor application. *Appl. Surf. Sci.* **462**, 353-361, doi:<https://doi.org/10.1016/j.apsusc.2018.08.089> (2018).

6 Amatucci, G. G., Badway, F., Du Pasquier, A. & Zheng, T. An Asymmetric Hybrid Nonaqueous Energy Storage Cell. *J. Electrochem. Soc.* **148**, A930, doi:10.1149/1.1383553 (2001).

7 Noori, A., El-Kady, M. F., Rahmanifar, M. S., Kaner, R. B. & Mousavi, M. F. Towards establishing standard performance metrics for batteries, supercapacitors and beyond. *Chem. Soc. Rev.* **48**, 1272-1341, doi:10.1039/C8CS00581H (2019).

8 Gangwar, J., Gupta, B. K., Kumar, P., Tripathi, S. K. & Srivastava, A. K. Time-resolved and photoluminescence spectroscopy of θ-Al2O3 nanowires for promising fast optical sensor applications. *Dalton Trans.* **43**, 17034-17043, doi:10.1039/C4DT01831A (2014).

9 Borchardt, L., Leistenschneider, D., Haase, J. & Dvoyashkin, M. Revising the Concept of Pore Hierarchy for Ionic Transport in Carbon Materials for Supercapacitors. *Adv. Energy Mater.* **8**, 1800892, doi:10.1002/aenm.201800892 (2018).

10 Yu, J. *et al.* Ultrahigh-rate wire-shaped supercapacitor based on graphene fiber. *Carbon* **119**, 332-338, doi:<https://doi.org/10.1016/j.carbon.2017.04.052> (2017).
